# Preliminary Evaluation of the Effectiveness of Perinatal Mindfulness-Based Well-Being and Parenting Programs for Low-Income New Mothers

**DOI:** 10.1007/s12671-023-02096-6

**Published:** 2023-03-07

**Authors:** Liliana J. Lengua, Stephanie F. Thompson, Rebecca Calhoun, Robyn B. Long, Cynthia Price, Ira Kantrowitz-Gordon, Lisa Shimomaeda, Paula S. Nurius, Lynn Fainsilber Katz, Jessica Sommerville, Cathryn Booth-LaForce, Anna Treadway, Alina Metje, Dannielle J. Whiley, Natasha Moini

**Affiliations:** 1 University of Washington, Psychology Box 351525, Seattle, WA 98195-1525 USA; 2 University of Toronto, Toronto, Canada

**Keywords:** Low income, Maternal mental health, Infant self-regulation, Perinatal, Prevention

## Abstract

**Objectives:**

This study examined specificity in the effects of three perinatal mindfulness-based prevention programs that differed in their timing (prenatal, postpartum) and target (maternal well-being, parenting). Effects on maternal mental health (depression, anxiety, resilience), mindfulness, and observed parenting, as well as observed, physiological, and mother-report indicators of infant self-regulation, were examined.

**Methods:**

The programs were evaluated in a racially and ethnically diverse sample of first-time mothers (*n* = 188) living in low-income contexts using intention-to-treat analysis. Mothers were assigned to a prenatal well-being, postpartum well-being, parenting, or book control group. Multi-method assessments that included questionnaire, observational, and physiological measures were conducted at four time points: during pregnancy (T1) and when infants were 2–4 months (T2), 4–6 months (T3), and 10–12 months.

**Results:**

Compared to the postpartum intervention and control groups, the 6-week prenatal well-being intervention was related to decreases in depressive symptoms during pregnancy but not postpartum, higher maternal baseline respiratory sinus arrhythmia (RSA), fewer intrusive control behaviors, and lower infant cortisol levels in the early postpartum period. Compared to all other groups, the postpartum parenting intervention was related to decreases in maternal anxiety and increases in responsive parenting. Some differential effects across programs might be due to differences in attendance rates in the prenatal (62%) vs. postpartum (35%) groups.

**Conclusions:**

The findings suggest that brief mindfulness-based well-being and parenting preventive interventions can promote maternal and infant mental health in families living in low-income, high-stress settings, particularly if accessibility can be enhanced.

**Preregistration:**

This study is not preregistered.

Low income and its associated stress have adverse effects on perinatal maternal well-being, parenting behaviors, and, in turn, infant development. Income-related stress and maternal mental health may alter the postnatal development of neurobiological systems underlying infant self-regulation through the shaping of the infant’s stress physiology during pregnancy. This is often referred to as prenatal programming of infant neurobiological systems (Berens et al., 2017; Somers & Luecken, 2022). Additionally, low income is related to disruptions in pre- and postnatal maternal well-being (Knitzer et al., 2008) and parenting behaviors (Paulson et al., 2006), which in turn may account for the effects of low income on infant developmental outcomes. Mindfulness-based interventions can reduce perinatal maternal stress and mental health problems (Burgdorf et al., 2019; Khoury et al., 2013), and support effective parenting (e.g., Shorey & Ng, 2021). Therefore, evaluation of mindfulness-based interventions that vary in their targets, such as maternal well-being or parenting, and that vary in their timing, in pregnancy or the early postpartum, might help clarify where and when to focus interventions to promote positive infant development in this at-risk context. This study examined outcomes specific to three purported mechanisms of low income on infant development underlying the effects of perinatal mindfulness-based interventions: prenatal programming, maternal mental health, and parenting.

Key indicators of neurobiologically based systems of self-regulation include regulation of the neuroendocrine stress responses system (HPA-axis reactivity), patterning of cardiac vagal outflow and respiration (respiratory sinus arrhythmia, RSA), and effortful control, reflecting executive-based regulation of emotion and behavior (Gartstein et al., 2013). Self-regulation provides a critical foundation for children’s long-term positive adjustment and school readiness (e.g., Ursache et al., 2012). However, children in low-income contexts tend to have lower self-regulation as early as the first year of life (Clearfield & Jedd, 2013). This may be due to early developmental alterations in the neurobiological underpinning of regulation relating to the stressors associated with living in low-income contexts (e.g., Thompson et al., 2009). There is a critical need to understand the factors that contribute to problems with self-regulation during infancy to prevent social, emotional, and behavioral problems and promote positive psychosocial adjustment and school readiness in young children growing up in poverty.

The pervasive effects of income-related adversity on children’s psychosocial adjustment may be accounted for by the impact of economic strain on parental well-being (resilience, mental health) and parenting behaviors, which in turn impact children’s well-being (Conger & Elder, 1997). Indeed, maternal well-being and parenting behaviors relate to indicators of infant self-regulation. Pre- and postnatal maternal mental health problems are associated with higher cortisol basal levels and reactivity in infants (e.g., Brennan et al., 2008), with the timing of symptoms impacting infant HPA functioning (Laurent et al., 2011). Additionally, parenting behaviors not only predict children’s HPA activity, but mediate the relations of income to children’s HPA-axis activity (Zalewski et al., 2012). Maternal mental health symptoms are also associated with higher child RSA reactivity (Ashman et al., 2008), and insensitive parenting is associated with difficulty in infants’ RSA recovery from stress (Ham & Tronick, 2006).

Low income is related to disruptions in pre- and postnatal maternal well-being (Knitzer, et al., 2008) and parenting behaviors (Paulson et al., 2006), which in turn may account for the effects of low income on infant developmental outcomes. Prenatal programming, maternal mental health, and parenting are potential mechanisms of these effects, each of which can be argued to have different effects on developmental outcomes (e.g., Van den Bergh et al., 2020).

The prenatal period has been implicated as a sensitive window in development during which maternal stress can confer lasting impairments to children (Seckl & Holmes, 2007) through structural and functional changes in the fetus, referred to as prenatal programming (Somers & Luecken, 2022). Animal studies have consistently demonstrated that stress experienced in gestation alters offspring’s organs, tissues, and systems and that these alterations result in lifelong observable changes to physiology, cognition, and behaviors (Weinstock, 2001). Published reviews of the human prenatal stress literature conclude that prenatal stress consistently relates to psychological maladjustment (Talge et al., 2007; Van den Bergh et al., 2005). Further, prenatally stressed children tend to show dysregulation in the neuroendocrine systems underlying adjustment (Harris & Seckl, 2011). Key indicators of neurobiologically based systems of self-regulation include regulation of the neuroendocrine stress response system (HPA-axis reactivity) and emotion regulation (respiratory sinus arrhythmia, RSA). Disruptions to these systems and their development might represent pathways of the effect of prenatal stress on children’s adjustment and account for the marked and enduring implications of prenatal experiences of stress.

Maternal mental health represents another potential mechanism of the effects of low income on infant development. It is well accepted that maternal mental health predicts children’s mental health (Goodman et al., 2011; Madigan et al., 2018; Stein et al., 2014). Effects of maternal mental health symptoms are evident as early as infancy on outcomes such as behavioral dysregulation, attachment, and cognitive/motor development (Irwin et al., 2020; Lyons-Ruth et al., 2002; Stein et al., 2014), and the effects of peripartum stress on child adjustment may persist through adolescence and into adulthood (Stein et al., 2014). Both prenatal and postpartum stress are relevant to infant adjustment. While prenatal stress has been defined in varied ways (c.f., Nast et al., 2013), the associations with infant adjustment are observed for symptoms of perinatal maternal depression (e.g., Gerardin et al., 2010) and maternal anxiety (e.g., Irwin et al., 2020), as well as negative life experiences (e.g., Bergman et al., 2007; Wurmser et al., 2006).

Parenting is an established mechanism of maternal stress and maternal mental health effects on child adjustment, and partially accounts for the effects of low income on infant development. In a meta-analysis of parenting interactions between mothers with depression and their young infants, mothers with depression were observed to be more irritable and hostile, to be less engaged, and to exhibit less warmth (Lovejoy et al., 2000), corresponding to an intrusive and overstimulating style of interaction or a withdrawn and understimulating style of interaction (Malphurs et al., 1996). The effects of postpartum depression on early parent-infant interactions are thought to be culturally universal and independent of socioeconomic status (Field, 2010). While it has been proposed that maternal anxiety is associated with parenting in infancy, with theoretical models suggesting an increased likelihood to engage in over-controlling parenting, the research on these associations has yielded inconsistent findings (see Van der Bruggen et al., 2008). Finally, mothers experiencing more negative life events are observed to be less responsive to their infant distress and engage in fewer behaviors that scaffold social and emotional growth (Crnic et al., 1983; McKelvey et al., 2002).

The specific effects of maternal mental health and parenting on infant adjustment are unclear, in part, due to their co-occurrence and their association with chronic adversity experienced by low-income families (Thompson et al., 2021), making it difficult to specify the extent to which these factors are contributing to infant well-being. However, interventions that differentially target specific aspects of maternal functioning can help to clarify these associations and identify targets for prevention. For example, sensitive, responsive parenting can offset the risk for self-regulation problems in very-low-birth-weight infants (Camerota et al., 2015). Further, when parents maintain effective parenting in contexts of low income and adversity, children demonstrate better self-regulation and social-emotional competence (Lengua et al., 2014). Mental health symptoms and parenting behaviors are more mutable relative to poverty, and mindfulness-based interventions show promise for decreasing stress and improving mental health symptoms among individuals faced with chronic stress (Creswell & Lindsay, 2014) and mental health (Grossman et al., 2004). They also show promising effects in supporting effective parenting in a low-income context (Lengua et al., 2021). Early preventive parenting interventions effectively enhance parenting behaviors and, in turn, infant adjustment outcomes (e.g., Bakermans-Kranenburg et al., 2003). We propose that supporting maternal well-being and effective parenting can promote self-regulation, positive psychosocial adjustment, and resilience in infants in low-income contexts.

The inter-relatedness of experiences of stress, maternal mental health problems, and parenting problems raises the question of what targets of intervention are more effective when considering both maternal and infant outcomes. Further, the timing of interventions could impact different aspects of maternal and infant well-being. In particular, mindfulness-based interventions hold promise for addressing stress management, mental health, and parenting, and were examined in this study as potentially impacting maternal and infant well-being.

Mindfulness-based approaches to reducing stress and increasing well-being during pregnancy are preferred by pregnant women relative to other treatment options (e.g., Dimidjian & Goodman, 2014). There have been a limited number of small RCTs examining mindfulness-based interventions carried out during pregnancy which are suggestive of changes to maternal anxiety, depression, and stress (Dhillon et al., 2017), but further evidence demonstrating effectiveness is needed. Existing studies have largely employed the Mindfulness-Based Cognitive Therapy with modifications for the perinatal period (e.g., Dimidjian et al., 2015) and the Mindfulness-Based Childbirth and Parenting (MBCP, Duncan & Bardacke, 2010; Duncan et al., 2017), which incorporates education on the psychobiological process of pregnancy and delivery as well as skills for relating to pain during childbirth. Preliminary evidence suggests that participation in mindfulness-based programs during pregnancy may confer lasting benefits into the early postpartum period, though few studies have tested long-term benefits (Dimidjian et al., 2016; Felder et al., 2018; Pan et al., 2019; Price et al., 2019). One study demonstrated decreases in distress related to a mindfulness-based childbirth class at a 12-month postpartum follow-up (Sbrilli et al., 2020).

Meta-analysis supports that mindfulness-based therapy is moderately to largely effective in improving psychological well-being, particularly in the domains of anxiety, depression, and stress (Khoury et al., 2013; van Agteren et al., 2021). The promise of benefits from mindfulness programs in the postpartum period has prompted national treatment guidelines to recommend such interventions (MacQueen et al., 2016; NCCMH, 2014). However, there is some evidence to suggest that these associations may not be robust to studies with small samples of women with pre-existing elevated levels of anxiety and depression (Shulman et al., 2018).

Mindfulness-based programs have also been implemented with the aim of improving parenting behaviors. It has been proposed that mindfulness or mindful parenting practices improve the parent–child relationship (Dumas, 2005; Duncan et al., 2009; Kabat-Zinn & Kabat-Zinn, 1998) and are associated with better child adjustment (e.g., Parent et al., 2016). However, there have been few systematic studies of mindful parenting interventions, and to our knowledge, fewer have targeted parents of infants. Initial studies of these interventions with older children are promising, showing parents engage in more positive parenting practices, experience less parenting stress, improve child compliance and capacity for attention regulation, and decrease internalizing and externalizing symptoms (Bögels et al., 2014; Felver et al., 2017; Lengua et al., 2021; Neece, 2014; Singh et al., 2010).

While a limited number of treatment studies have specifically targeted perinatal maternal mental health for women living in the context of low income (c.f. Beardslee & Knitzer, 2004), there is a dearth of research examining the feasibility and acceptability of mental health therapies modified for the perinatal period that are available for women living in low-income contexts. Existing adaptations include service delivery through community primary care or home visitation, which are cost intensive and difficult to scale (O’Mahen et al., 2013). In addition, there are substantial practical barriers to accessing interventions, including caregiving responsibilities, having multiple jobs, lacking access to affordable childcare and transportation, and housing insecurity. Addressing these barriers is critical to supporting families living in the context of low income.

This study evaluated the potential differential effects of three brief mindfulness-based well-being and parenting programs on maternal mental health, parenting, and infant well-being in a sample of first-time mothers and infants experiencing economic disadvantage. Given that parents in low-income contexts experience significant stress and adversity and, as a result, are at elevated risk for mental health and parenting problems, it was expected that mindfulness-based stress management and parenting interventions could reduce this risk and support both parent and infant well-being. Mothers were assigned to one of three programs or a book control group: (1) a prenatal mindfulness-based stress-management program adapted from MBCP (Table 3), which was expected to support better mental health in pregnant mothers, thereby potentially reducing the effects of prenatal programming of stress manifested in infant HPA-axis regulation; (2) a similar mindfulness-based stress-management program delivered in the postpartum period, which was expected to promote maternal well-being which could potentially socialize infant self-regulation; (3) a parenting program infused with mindfulness practices, adapted from the Social-Emotional Competence for Children and Parents (SEACAP, Lengua et al., 2021) that was designed to promote more sensitive, responsive parenting practices that are shown to support the development of executive functioning, higher RSA, and HPA-axis regulation; and (4) a control group that received two high-quality books about pregnancy and infant development. The programs were distinct in that the two programs adapted from MBCP included mindfulness practices to support emotion regulation and stress management, with little or no attention to parenting behaviors, whereas the SEACAP program included behavioral parenting practices supported with informal mindfulness practices. Differential effects of the three programs on a range of maternal and infant outcomes could specify the effects of prenatal programming, parent mental health, and parenting on different infant self-regulation systems. Further, if differential effects of targeting maternal well-being or parenting can be identified, the optimal timing and target of intervention to prevent adverse outcomes and promote positive psychosocial adjustment in children growing up in economic disadvantage can be specified.

The goals were to evaluate relatively brief interventions to increase feasibility, accessibility, and participation by new mothers and to assess the potential for differential effects of the interventions that varied on timing and target. The content was designed to be culturally responsive and trauma sensitive. To standardize the amount of contact with participants, all programs were 6 weeks in duration with 2-hr sessions each week, for a total of 12 hr of intervention. The three programs differed on their timing (prenatal vs. postpartum) and target (maternal well-being vs. parenting behaviors) to address the following questions: (1) Does a prenatal mindfulness-based childbirth class improve maternal mental health and well-being during pregnancy? (2) Do any effects of a prenatal mindfulness-based childbirth class have continued benefits to maternal mental health, well-being, and parenting behaviors in the early postpartum period? (3) Do postpartum programs that target maternal mental health/well-being vs. parenting behaviors have differential effects on maternal mental health, parenting behaviors, and infant well-being? (4) Are program effects evident at a 6-month follow-up assessment?

## Method

### Participants

Participants were 188 first-time mothers recruited during their second trimester of pregnancy. Participants were eligible if they were at least 18 years old and had a household income less than 200% of the U.S. Federal Poverty Level, operationalized as less than $45,000 annual income for a family of three. Participants indicated whether they were fluent in the English language to the extent that they were able to participate in assessments and classes conducted in English. Exclusion criteria included women who (1) were beyond the 33^rd^ week of pregnancy; (2) had self-reported addiction to alcohol or other substances; (3) had self-reported mental illness which caused hallucinations or altered perception of reality; (4) had previously given birth; (5) were under age 18; and (6) were pregnant with twins or multiples. Recruitment was conducted at obstetrics, midwifery, and public health clinics in the greater Seattle metropolitan area.

Mothers’ average age was 26.44 (*SD* = 5.95, range = 18–43). Mothers were racially and ethnically diverse: 35% African-American/Black, 12% Asian American, 2% Hawaiian/Pacific Islander, 17% Latinx/Hispanic, 6% Native American/American Indian, 25% White, and 3% multiracial participants, with 22% identifying as immigrants and 4% as refugees. Families’ income included 36% < $14,600, 22% $14,600–$25,790, 25% $25,791–$37,010, and 17% > $37,011. The sample reported high levels of adverse childhood experiences (*M* = 3.13, *SD* = 2.53), with 42% of the sample reporting having 4 or more adverse childhood experiences. There were no differences in these participant demographics based on intervention group assignment.

### Procedure

All procedures were approved by the university’s human subjects institutional review board, and informed, signed consent was obtained from all mothers prior to participating in study procedures. Participants were assessed a total of 4 times (with participants in the prenatal condition and a subset of all other participants completing a late pregnancy assessment that served as a post-test after the prenatal intervention). All participants were first assessed during pregnancy and then assigned to one of the 4 groups (prenatal well-being [*n* = 46], postpartum well-being [*n* = 48], postpartum parenting [*n* = 53], control [*n* = 39]) based on their estimated due date so that participants in each group had due dates close in time. As recruitment occurred on an ongoing basis over a 2-year period, participants were assigned to groups on a rolling basis based on their estimated due dates, staggering the interventions. Participants assigned to the control group either had due dates that did not align with the group clusters or were located in remote geographies. Control group participants received two high-quality pregnancy and infant development and parenting books.

Assessments took place in offices on the university campus when mothers were pregnant (T1), which served as the prenatal intervention pretest; when infants were 2–4 months (T2), which served as the postpartum intervention pretest; when infants were 4–6 months (T3), which served as the postpartum intervention post-test; and when infants were 10–12 months (T4), which served as the follow-up assessment (Table 1). T1 assessments were completed by 188 participants, T2 assessments were completed by 168 participants, and T3 and T4 assessments were completed by 160 and 162 participants, respectively.

**Table 1:** Assessment and intervention timeline for each study group

Group	T1 Prenatal	Intervention	T1b Prenatal Post-test	T2 Infants 2–4 months	Intervention	T3 Infants 4–6 months	T4 Infant 10–12 months
Prenatal well-being	**✓**	**✓**	**✓**	**✓**		**✓**	**✓**
Postpartum well-being	**✓**			**✓**	**✓**	**✓**	**✓**
SEACAP parenting	**✓**			**✓**	**✓**	**✓**	**✓**
Book control	**✓**			**✓**		**✓**	**✓**

Mothers who participated in the prenatal intervention, along with a randomly selected subset of participants not receiving the prenatal intervention, also completed an abbreviated assessment immediately after the completion of the prenatal intervention (T1b), which served as the prenatal intervention post-test. This assessment was not part of the original study design but was added partway through the study in order to test for effects of the prenatal program during pregnancy. Assessments included maternal reports on questionnaires assessing the family context and experiences of stress, mindfulness, well-being, mental health and infant temperament, observational measures of parent-infant interactions, and physiological assessments including maternal and infant RSA and cortisol. Mothers were compensated $90 for the first assessment, and compensation increased by $10 for each subsequent assessment. In addition, parking and transportation were provided. Participants received $10 for each intervention session they attended.

The three interventions were offered in person in community settings that were located to facilitate parent participation. Each of the 3 interventions had different co-facilitators, and those facilitators offered their respective intervention multiple times to 7–14 mothers. Facilitators were trained and provided ongoing supervision by the respective intervention developers. Facilitators tracked the content delivered in each session to demonstrate fidelity to each intervention. The percentages of program content delivered for the three programs were 99% for the prenatal well-being, 97% for postpartum well-being, and 94% for the SEACAP parenting program.

Each intervention consisted of six 2-hr weekly sessions and two optional half-hour individual coaching sessions and were facilitated by two facilitators. Rates of attendance are reported in Table 2. Attendance was lower in the postpartum groups compared to the prenatal group. In the prenatal group, 84% of participants attended one or more sessions, with 67% of participants attending three or more sessions. In both postpartum groups, 48% of participants attended at least one session with 35% of participants in the postpartum well-being groups and 38% of participants in the SEACAP parenting groups attending three or more sessions (Table 2). Those who attended reported high satisfaction with the programs: 96% agreed or strongly agreed that they were satisfied with skills learned, 97% found the information useful and were comfortable in the group environment, and 94% indicated they were satisfied with the program overall. See Table 3 for the content of the interventions.

**Table 2:** Number of participants attending 0–6 sessions for each intervention group, excluding individual sessions

	Number of sessions attended						
Intervention group	0	1	2	3	4	5	6
Prenatal well-being	7	4	4	3	7	8	13
Postpartum well-being	25	3	3	3	5	4	5
SEACAP parenting	27	3	2	3	6	7	5
Book control	39

**Table 3:** Primary themes and topics in NEW Moms Connect interventions

Session #	Primary themes and topics covered		
	Prenatal maternal well-being	Postpartum maternal well-being	Postpartum parenting
Class 1	Mindfulness introduction and its relationship to stress and change Pregnancy, birth, and parenting as a time of change and transition	Mindfulness introduction and its relationship to stress and change Becoming a parent as a time of change and transition	Introduce infancy as a sensitive period and core parenting skills: being present, warm, consistent, and sensitive. Encourage consistent responding to baby cues
Class 2	Practice breath awareness Importance of perception in how we relate to stress, physical pain, and pleasant/unpleasant moments Physiology of childbirth and relationship to mindfulness	Practice breath awareness, body scan Importance of perception in how we relate to stress, emotional pain, and pleasant/unpleasant moments Physiology of stress and relationship to mindfulness	Psychoeducation on emotions, the potential for emotions to interfere with effective parenting, and the importance of regulating emotions Strategies for scaffolding sustained attention and infant play
Individual 1	Sensory awareness through breath Mental intention/attention and mindfulness	Sensory awareness through breath Mental intention/attention	Reinforce parent wisdom Distress tolerance implementation
Class 3	Practice mindful movement Explore mindfulness in stressful events, physical and emotional Demonstrate mechanisms of labor and positions for birth	Practice mindful movement Explore mindfulness in stressful events, physical and emotional Discuss judging, comparing, and the storytelling mind	Discuss strategies for accessing internal wisdom Develop a distress tolerance plan to interrupt emotion dysregulation, the “Parent Time Out” as wise parenting
Class 4	Practice body scan meditation Discuss the illusion of control and acknowledgement of “what is,” and the importance of flexibility in coping	Practice loving kindness for parenting Discuss fear as future thinking and self-compassion practices Practical skills for self-care when caring for a new baby	Introduce the five senses as a tool for being present and for enriching infant experiences Information on promoting interaction and scaffolding language development
Individual 2	Mindfulness practice and pregnancy check-in Body scan: overview, purpose	Mindfulness practice and parent check-in Body scan: overview, purpose	Create an individualized, consistent bedtime routine. Reinforce responsiveness and language through play
Class 5	Discussion of fear as future thinking Self-compassion introduction Practical skills for self-care when caring for a new baby	Practice awareness of sound The illusion of control and acknowledgement of “what is,” the importance of flexibility Mindfulness and working with resistance	Introduce skills for promoting self-compassion Discuss infant sleep and consistency in relation to infant sleep hygiene
Class 6	Newborn needs/breastfeeding Using mindfulness to identify needs of self and baby	Defining/discussing resilience Using mindfulness to identify strengths and resources in early parenting	Note the physical experiencing of emotions, and the potential to model emotion regulation

The prenatal intervention was adapted from MBCP with permission from its author by an individual trained and certified in offering MBCP and our research team

### Measures

Assessments included measures of maternal mental health, well-being, mindfulness, and parenting, and observed, physiological, and maternal-report indicators of infant self-regulation.

*Maternal mental health and well-being* were assessed using measures of pregnancy-specific anxiety, depression and anxiety symptoms, resilience, and baseline RSA.

*Pregnancy-specific anxiety* was assessed at T1 using the 10-item Prenatal Pregnancy Anxiety Scale (Rini et al., 1999). Pregnant participants reported on their confidence and worries regarding their baby’s development as well as their pregnancy and upcoming labor and delivery*.* Internal consistency as measured by Cronbach’s alpha (*α*) was 0.84, and scale reliability as indicated by McDonald’s omega (*ω*) was 0.84. *Depressive symptoms* were assessed at all time points using the 20-item Center for Epidemiologic Studies Depression Scale (CES-D; Radloff, 1977) which assesses the frequency of symptoms experienced in the past week for depressed affect, somatic activity, and interpersonal relations. Studies of the scale’s psychometrics support its longitudinal invariance, that is, that changes in symptom scores on this scale reflect true symptom change (Ferro & Speechley, 2013). The internal consistencies for this study were *α* = 0.87–0.93 and scale reliabilities were *ω* = 0.87–0.93. *Anxiety symptoms* were assessed at all time points using the 7-item Generalized Anxiety Disorder Scale (GAD-7; Spitzer et al., 2006) which assessed core symptoms of generalized anxiety disorder. The scale has evidenced good reliability and validity in general population studies (Löwe et al., 2008). The internal consistencies were *α* = 0.85–0.90 and scale reliabilities were *ω* = 0.85–0.90. Depression and anxiety demonstrated moderate to high stability across time points (depression *r* = 0.54–0.73; anxiety *r* = 0.45–0.67).

*Mindfulness and self-compassion* were assessed at T1, T2, and T3. The 24-item Five Facet Mindfulness Questionnaire-Short Form (FFMQ-SF; Baer et al., 2006) was used to capture the extent to which individuals practice observing, describing, and acting with awareness, non-judgmentally and non-reactively. The FFMQ Short Form has been found to have good reliability and validity (Bohlmeijer et al., 2011). The internal consistencies for the current study were *α* = 0.81–0.85, and scale reliabilities were *ω* = 0.82–0.85. The 12-item Self-Compassion Scale–Short Form (SCS-SF; Raes et al., 2011) was used to assess the degree to which participants adopted a non-judgmental understanding of pain, the universality of the human experience, and the worthiness of all people—oneself included—of compassion. Six 2-item factors spanned self-kindness, common humanity, and mindfulness. The SCS-SF demonstrates adequate validity and reliability (e.g., Hayes et al., 2016; Kelly et al., 2013). The internal consistency reliabilities for the current study were *α* = 0.86–0.88 and McDonald’s omega was *ω* = 0.86–0.87. Mindfulness and self-compassion demonstrated high stability across time points (mindfulness *r* = 0.61–0.79; self-compassion *r* = 0.65–0.86).

In addition, at T4, participants were asked the extent to which they used specific practices that were included in one or more of the interventions, including focusing on the breath, body scan, yoga or mindful stretching, mindfully being with baby, and self-compassion. Participants indicated whether they used the practices *not at all* (0) to *almost every day* (5). If they indicated that they did not attend any classes, they were assigned a value of 0. Excluding the control group, participants reported an average of 2.42, corresponding approximately *once or twice a month* (*SD* = 1.64) and a range of 0–5.

*Resilience* was assessed at all time points as respondents’ self-perceived ability to recover from stress using the 6-item Brief Resilience Scale (BRS; Smith et al., 2008). The measure demonstrates adequate internal consistency and convergent and discriminant validity, including distinctiveness from constructs of depression, anxiety, and stress (Kyriazos, 2018). The internal consistency reliabilities were *α* = 0.84, and the scale reliabilities were *ω* = 0.84 at all time points. The BRS demonstrated moderate to high stability across time points (*r*s = 0.67–0.77).

*Observed parenting* was assessed at T2 and T3 from video-recorded parent-infant interactions during the still-face paradigm (SFP; Tronick et al., 1978). This included four segments: naturalistic play (3 min) in which the mother was instructed to play with her infant as she normally would; free play (2 min) in which the mother was again instructed to play as she normally would while both mother and infant were wearing wires and belts to gather cardiac and respiration data for RSA; still face (2 min, but ended after 30 s of extreme infant distress) during which the mother was asked to adopt a neutral face and not respond to the bid of her infant; and reunion (5 min) during which the mother was instructed to resume normal interactions with her infant. The SFP is a versatile assessment that may vary slightly, for example, in the duration of episodes, and has been found to relate to both parenting behaviors and predict future adaptation of offspring (Mesman et al., 2009).

Parent behaviors were rated from video recordings by trained research assistants after attaining reliability. Parent scaffolding, responsiveness, negative affect, and negative control were coded based on an infant adaptation of rating scales (Lengua et al., 2014) based on established systems for coding parent–child interactions (Cowan & Cowan, 1992; Lindahl & Malik, 2014; Rubin & Cheah, 2000). The *scaffolding* code assessed the overall level of parental guidance that facilitated and enhanced infants’ play, or well-timed behaviors that facilitated the focus, engagement, and enjoyment of the infant in play. *Responsiveness* referred to the degree to which mothers noticed and responded in an appropriate and timely fashion to infant distress. *Negative affect* captured the frequency and intensity of verbal and nonverbal behavior that was negative, dismissive, or hostile to the infant. *Negative control* quantified the degree to which mothers exerted inappropriate, ill-time, or excessive behaviors that were intrusive or inappropriately stimulating (causing dysregulation). Dimensions were coded in 30-s epochs on a 6-point scale during the interactive episodes of the SFP. Epochs were averaged across episodes to calculate a global score for each dimension. Inter-rater agreements as indicated by the intra-class correlation (ICC) were moderate to high for each dimension (scaffolding 0.83, responsiveness 0.58, negative affect 0.87, negative control 0.85), with alphas for the scores averaged across epochs of 0.82 for scaffolding, 0.64 for responsiveness, 0.76 for negative affect, and 0.74 for negative control. Omegas were 0.81 for scaffolding, 0.72 for responsiveness, 0.78 for negative affect, and 0.82 for negative control.

*Observed infant emotion regulation*: Infant self-soothing, positive affect, and negative affect were coded in 30-s epochs using a 6-point scale, with aggregate scores capturing the overall level of each dimension during all episodes of the SFP. *Self-soothing* captured the extent of self-comforting behaviors such as auto-manipulative thumb or finger sucking. *Infant positive affect* assessed the positive quality of the infant’s expressions and overall agreeableness and enjoyment of the interaction. *Infant negative affect* defined the frequency and intensity of displays of overstimulation, frustration, or distress in the infant. ICCs were 0.90 for self-soothing, 0.82 for positive affect, and 0.96 for negative affect. Internal consistency of the codes averaged across epochs as indicated by alpha was 0.70 for positive affect, 0.59 for negative affect, and 0.79 for self-soothing. Scale reliabilities as indicated by omega were 0.72 for positive affect, 0.68 for negative affect, and 0.82 for self-soothing.

*Physiological measures of infant regulation* included baseline RSA, and cortisol baseline and reactivity. RSA was recorded using Biopac MP150 equipment with Acqknowledge software. Data were collected at 1 kHz sampling rate and were loaded into custom Matlab software to detect R-wave peaks and screen/correct for artifacts. R-wave peaks were initially identified from local maxima of the ECG waveform that lay above a voltage threshold set to the 98th percentile of all the ECG data points. Inter-beat intervals (IBIs) were plotted as a function of the time of the R-peak that concluded each corresponding interval. False or missed R-peaks and spurious IBI data were excluded if transient, high-frequency interference had substantially distorted the shape of the ECG wave components and generated additional peaks, which was identified by visual inspection. Clean intervals less than 20 s in duration were also excluded to maintain a minimum of 0.05 Hz frequency resolution for spectral analysis of IBI data.

Baseline RSA reflects the average RSA value of epochs for a 3-min period during which the mother held her infant facing her on her lap, and she was instructed to sit quietly and not play or talk with her baby. For each behavioral assessment, the mean and standard deviation of the IBIs were computed (milliseconds); the reciprocals of these IBI values were used to compute mean and standard deviation of heart rate (beats per minute). To compute RSA, the IBI data were interpolated onto a time scale that sampled at 2 Hz and then Fourier transformed. RSA was defined as the log of average power density from frequency components in the band falling between 0.33 and 0.42 Hz (Litvack et al., 1995). This band corresponds to the range of respiration rates reported by Behrman et al. (2002) for infants 6–12 months.

HPA-axis activity was assessed by cortisol baseline and reactivity levels. Cortisol levels were assayed from saliva samples collected by trained experimenters using infant sorbettes placed in the corner of the infant’s mouth or under the infant’s tongue for approximately 90 s or until an adequate saliva volume was obtained. Baseline measures were obtained after introductions and the informed consent procedure was complete, approximately 20 min after the family’s arrival. Reactivity measures were obtained 20 min after the SFP was complete. Samples were immediately frozen and stored at − 70 °C. Samples transported to the university’s School of Nursing Biochemical Laboratory for processing without undergoing a thaw cycle, where they were stored at − 70 °C until extraction. All sample tubes were thawed to room temperature and centrifuged at 1000 rpm for 10 min in order to separate the saliva from the collection swab. The cleared eluant was then transferred to a 1.6-mL Eppendorf tube and stored at − 70 °C until testing for cortisol. Prior to the assay, each sample was subjected to another centrifugation step of 5000 rpm for 5 min in order to separate out small particulates and residual mucin. To test for the presence of salivary cortisol, 25 μL of saliva from each sample was transferred into each of two wells, producing duplicate samples for each assay; sample values were then averaged. The concentration of cortisol in each sample was extrapolated from a standard curve generated in each test plate, and the results were averaged in order to give an adjusted result. Samples were assayed using the High-Sensitivity Cortisol Salivary Enzyme Immunoassay Kit provided by Salimetrics LLC (State College, PA). The sensitivity of this kit ranges from 0.012 to 3.000 µg/dL. All samples from the same subject for each set of saliva were included in the same assay batch to minimize inter-assay within-subject variability.

Assay results that were > 3 SDs above the mean were winsorsized to 3 SD. As is common with cortisol data, values were positively skewed, and log transformations were applied prior to analysis. Potential covariates including time of collection, what the infant was last fed (breastmilk or formula), when the infant was last fed, when the infant last woke from a nap, maternal medications (if breastfeeding), and infant illnesses and medication were examined in relation to the cortisol values. There were no significant associations of baseline or reactivity values with potential covariates. Validity of the measure of salivary cortisol as an indication of infant regulation is suggested by associations of baseline (*r* = 0.18, *p* = 0.11) and reactivity (*r* =  − 0.26, *p* = 0.02) levels in infancy with maternal reports of behavior problems at age 24–42 months.

*Infant temperament* was assessed at T3 and T4 by mothers’ reports on the 37-item Infant Behavior Questionnaire Revised – Very Short Form (Putnam et al., 2014) which produced scores on infants’ negative affect and effortful control. Negative affect assesses infant crying, fussing, and distress. The alphas in this study were 0.77 and 0.75, and the omegas were 0.68 and 0.69. Effortful control assesses infant attention, soothability, and enjoyment of low-intensity activities. The alphas were 0.66 and 0.73 and the omegas were 0.63 and 0.75. These were utilized as indicators of infant emotion regulation.

*Covariates* examined included family income, economic strain, maternal age, educational attainment, housing instability, adverse childhood experiences (ACEs), pregnancy concerns and complications, planned pregnancy, and number of program sessions attended. Participants reported on family income from all sources on a 14-point Likert scale (1 = $14,570 or less, 2 = $14,571–$18,310, 3 = $18,311–22,050, etc.; *M* = 5.23, *SD* = 3.81). Mothers reported on their age, educational attainment, whether they had received mental health treatment in the past, whether they currently have a mental health diagnosis, whether they were told by a physician that there were concerns or complications related to their pregnancy, and whether the pregnancy was planned. Participants reported on their financial insecurity on seven items assessing the degree to which they had enough money to afford living essentials (e.g., home, food, medical care, etc.). Scores were reversed so that higher scores represented higher insecurity. Housing instability was indicated by mothers reporting that they were temporarily living with others or were homeless. Mothers reported their history of adverse childhood experiences (ACEs) using the 10-item ACEs Questionnaire (Felitti et al., 1998) which assessed childhood trauma in three main categories: abuse (emotional, physical, sexual), neglect (physical, emotional), and household challenges (family members who were diagnosed with mental illness or attempted suicide, had been incarcerated, had misused alcohol or drugs; parental loss through divorce, separation, or death; domestic violence). Scores are the number of experiences endorsed. Participants reported on the frequency of their use of mindfulness practices taught in at least one of the interventions (e.g., body scan, yoga/mindful stretching, loving kindness) indicating using them not at all, once or twice a month, once a week, several times a week, or almost every day.

### Data Analyses

Differences across the four study groups were examined using multiple regression models tested using full information maximum likelihood estimation (FIMLE) to address missing data. First, covariates were identified based on significant associations with group assignment, missingness, and/or T1 levels of the outcome variables. Next, multiple regressions were used to test whether:

1. the prenatal program was related to changes in maternal mental health and well-being from pretest to post-test assessments by examining program effects on the post-test levels of maternal mental health immediately following the prenatal program (T1b-post-test), controlling for pretest levels and covariates (T1). This was tested by entering in the regression a dichotomous variable indicating participation in the prenatal program (1, *n* = 46) compared to those who did not (0, a subset of the sample was administered T1b-post-test measures, *n* = 23; this subset included randomly selected participants from the other 3 groups).
2. the prenatal program demonstrated continued benefits to maternal mental health, well-being, mindfulness, parenting, and infant self-regulation at the early post-partum assessment (T2) compared to all other groups. This was tested by entering in the regression a dichotomous variable indicating participation in the prenatal program (1, *n* = 46) compared to those who did not participate in the prenatal program (0, *n* = 140, i.e., all other participants), controlling for the corresponding T1 variables and covariates.
3. the prenatal and postpartum programs were differentially related to changes in maternal mental health, well-being, parenting, and infant self-regulation from pretest (T2) to post-test (T3) assessments by examining program effects on post-test levels controlling for pretest levels. This was tested by entering in regressions three dichotomous variables indicating participation in the prenatal well-being, postpartum well-being, and parenting groups, thereby comparing each intervention group to each other and the control group, controlling for the corresponding T2 variables and covariates.
4. the three groups differed from each other and from the control group in maternal mental health, well-being, and parenting and infant outcomes at the follow-up assessment when infants were 10–12 months (T4) by comparing each intervention group to each other and the control group, controlling for the corresponding T2 variables and covariates.

## Results

Means and *SD*s for maternal and infant outcome variables are reported in Tables 4 and 5, respectively. Covariates were identified based on significant correlations with group assignment, missingness, or T1 outcomes. Variables examined included family income, economic strain, maternal age, education, housing instability, ACEs, pregnancy concerns and complications, and number of program sessions attended. Comparing participants based on group assignment, the control group reported higher economic strain and housing instability. Economic strain, housing instability, and number of sessions attended were significantly associated with missingness or were consistently associated with T1 variables. Economic strain and housing instability were included as covariates in all analyses. All analyses were conducted excluding and including number of sessions attended to ascertain the impact of attendance on the results.

**Table 4:** Means (standard deviations) of maternal outcomes for each study group at each time point

Maternal outcomes	Prenatal well-being				SEACAP				Postpartum well-being				Control			
	T1	T2	T3	T4	T1	T2	T3	T4	T1	T2	T3	T4	T1	T2	T3	T4
Mental health																
Pregnancy anxiety	18.13 (4.78)				17.70 (4.79)				19.58 (6.42)				18.58 (5.28)			
Depression	13.47 (7.94)	13.68 (11.08)	15.11 (10.81)	13.31 (9.01)	14.23 (8.15)	13.28 (10.98)	12.59 (9.07)	14.42 (9.98)	14.35 (9.13)	13.89 (12.73)	13.40 (10.15)	13.23 (10.65)	16.11 (10.10)	15.45 (9.83)	15.57 (10.32)	15.06 (10.45)
Anxiety	5.39 (4.14)	5.83 (4.85)	5.83 (4.54)	5.19 (3.79)	6.28 (3.77)	5.89 (5.13)	5.41 (4.61)	6.48 (5.04)	6.69 (4.46)	6.67 (5.13)	6.51 (4.88)	6.07 (4.86)	6.42 (4.62)	5.75 (5.15)	5.40 (4.54)	5.47 (5.29)
Resilience	21.63 (4.18)	22.20 (4.10)	22.14 (4.57)	22.47 (4.11)	20.68 (4.85)	21.38 (4.70)	21.34 (5.03)	21.67 (4.79)	21.48 (4.33)	22.22 (5.10)	21.64 (4.48)	22.70 (5.12)	19.79 (3.33)	20.19 (4.31)	19.27 (3.10)	20.15 (3.88)
Baseline RSA		5.92 (1.24)	5.65 (1.13)			5.15 (1.23)	5.39 (1.21)			5.43 (1.30)	5.20 (1.42)			5.14 (0.90)	5.15 (0.90)	
Mindfulness																
Mindfulness	85.79 (10.38)	86.86 (11.22)	87.62 (12.50)		84.94 (10.26)	86.26 (12.23)	86.00 (14.76)		83.38 (11.23)	85.84 (12.62)	84.85 (13.15)		81.54 (11.74)	82.75 (11.28)	80.84 (11.63)	
Self-compassion	43.32 (8.62)	43.38 (7.28)	42.31 (7.19)		41.11 (8.96)	41.48 (9.24)	40.78 (9.99)		39.60 (10.10)	42.55 (10.72)	42.73 (9.82)		39.85 (7.17)	40.88 (9.09)	40.10 (8.98)	
Observed parenting																
Scaffolding		3.19 (0.68)	3.07 (0.60)			3.36 (0.58)	3.18 (0.51)			3.21 (0.70)	3.21 (0.50)			2.95 (0.74)	2.64 (0.74)	
Responsiveness		3.18 (1.10)	2.53 (0.97)			3.17 (1.06)	3.22 (0.91)			2.80 (1.21)	2.95 (1.17)			2.96 (1.38)	2.47 (1.16)	
Negative affect		0.20 (0.30)	0.36 (0.43)			0.26 (0.29)	0.24 (0.28)			0.23 (0.33)	0.29 (0.40)			0.23 (0.30)	0.39 (0.42)	
Negative control		1.86 (0.79)	2.09 (0.96)			2.18 (0.93)	2.08 (0.88)			2.18 (0.82)	2.39 (1.05)			2.11 (0.87)	2.05 (1.10)	

T1 is prenatal, T2 is when infants were 2–4 months, T3 is when infants were 4–6 months, and T4 is when infants were 10–12 months

**Table 5:** Means (standard deviations) of infant outcomes for each study group at each time point

Infant outcomes	Prenatal well-being				SEACAP				Postpartum well-being				Control			
	T1	T2	T3	T4	T1	T2	T3	T4	T1	T2	T3	T4	T1	T2	T3	T4
Observed emotion regulation																
Self-soothing		2.53 (1.12)	3.04 (1.17)			2.44 (1.17)	2.58 (1.22)			2.23 (1.26)	2.49 (1.16)			2.10 (1.21)	3.10 (1.14)	
Positive affect		1.81 (0.82)	2.12 (0.71)			1.88 (0.65)	2.08 (0.60)			1.79 (0.63)	2.11 (0.64)			1.43 (0.48)	1.94 (0.48)	
Negative affect		1.63 (0.88)	1.25 (0.64)			1.92 (0.79)	1.56 (0.81)			1.62 (0.80)	1.40 (0.73)			1.90 (0.87)	1.49 (0.74)	
Physio regulation																
Baseline RSA		3.45 (1.14)	3.42 (0.97)			3.52 (1.35)	3.68 (0.94)			3.28 (1.51)	3.46 (0.97)			3.40 (1.08)	3.39 (0.90)	
HPA-axis Baseline		− 1.03 (0.42)	− 0.69 (0.49)			− 0.87 (0.46)	− 0.80 (0.44)			− 0.89 (0.36)	− 0.96 (0.50)			− 0.80 (0.34)	− 0.86 (0.43)	
HPA-axis Reactivity		0.23 (0.56)	0.16 (0.63)			0.05 (0.49)	0.03 (0.59)			− 0.03 (0.35)	− 0.04 (0.47)			0.21 (0.52)	− 0.06 (0.47)	
Temperament																
Effortful control			65.56 (9.43)	60.63 (8.38)			66.52 (8.22)	63.50 (9.21)			63.51 (10.44)	59.69 (10.72)			61.77 (10.44)	57.89 (9.03)
Negative affect			41.67 (11.83)	48.46 (12.49)			43.02 (9.07)	49.63 (9.64)			41.00 (14.02)	47.65 (13.92)			42.67 (15.02)	47.83 (13.60)

T1 is prenatal, T2 is when infants were 2–4 months, T3 is when infants were 4–6 months, and T4 is when infants were 10–12 months

Results of regression analyses testing study hypotheses are presented in Table 6. Regression analyses were used to test whether the prenatal program was related to changes in maternal mental health, well-being, and mindfulness during pregnancy (T1b, prenatal post-test) controlling for T1 levels of the corresponding variables and covariates. There was a significant effect of participating in the prenatal well-being group on depression and a trend toward an effect on anxiety, with the intervention group demonstrating lower levels of each. There were no differences on pregnancy anxiety, resilience, mindfulness, or self-compassion.

**Table 6:** Standardized regression coefficients (standard errors) comparing interventions to each other and the control group

	T1b		T2		T3				T4			
	PRE	COV	PRE	COV	PRE	SEA	PPW	COV	PRE	SEA	PPW	COV
Maternal outcomes												
Mental health												
Pregnancy anxiety	− 0.104 (0.075)											
Depression	− 0.217* (0.079)	3	0.027 (0.016)	2	0.042 (0.069)	− 0.058 (0.068)	0.000 (0.068)	2	− 0.055 (0.071)	0.014 (0.071)	0.006 (0.071)	
Anxiety	− 0.152^t^ (0.082)		0.029 (0.065)	1	0.089 (0.080)	− 0.206* (0.079)	0.142^t^ (0.079)		− 0.011 (0.084)	0.107 (0.084)	0.079 (0.083)	
Resilience	0.068 (0.068)		0.025 (0.052)		0.116^t^ (0.069)	0.099 (0.068)	0.054 (0.067)		0.116 (0.072)	0.091 (0.071)	0.117 (0.071)	
Baseline RSA			0.215* (0.087)		− 0.052 (0.127)	0.024 (0.127)	− 0.126 (0.124)					
Mindfulness												
Mindfulness	− 0.071 (0.081)		0.029 (0.056)	3	0.073 (0.067)	0.034 (0.067)	0.045 (0.065)					
Self-compassion	− 0.074 (0.076)		− 0.013 (0.056)		− 0.014 (0.058)	− 0.011 (0.057)	0.020 (0.057)					
Observed parenting												
Scaffolding			− 0.040 (0.083)		0.237* (0.111)	0.305** (0.109)	0.347** (0.105)	2				
Responsiveness			0.061 (0.103)		0.065 (0.152)	0.345* (0.143)	0.244^t^ (0.142)					
Negative affect			− 0.053 (0.083)		− 0.067 (0.122)	− 0.224^t^ (0.120)	− 0.169 (0.119)					
Negative control			− 0.162* (0.081)		0.049 (0.123)	0.020 (0.122)	0.145 (0.119)					
Infant outcomes												
Observed emotion regulation												
Self-soothing			0.077 (0.088)		− 0.014 (0.129)	− 0.203 (0.128)	− 0.180 (0.126)	1, 3				
Positive affect			0.023 (0.087)		0.026 (0.129)	− 0.008 (0.131)	0.016 (0.127)					
Negative affect			− 0.087 (0.087)		− 0.072 (0.129)	0.079 (0.128)	0.021 (0.127)					
Physio regulation												
Baseline RSA			0.017 (0.095)	1, 3	− 0.040 (0.139)	0.079 (0.140)	− 0.022 (0.136)					
HPA-axis baseline			− 0.180* (0.085)		0.179 (0.127)	0.060 (0.127)	− 0.085 (0.126)					
HPA-axis reactivity			0.154^t^ (0.086)		0.110 (0.116)	0.074 (0.115)	0.047 (0.115)					
Temperament												
Effortful control					0.106 (0.104)	0.170^t^ (0.103)	0.020 (0.102)	1	0.066 (0.102)	0.196* (0.099)	− 0.012 (0.100)	1
Negative affect					− 0.009 (0.107)	0.039 (0.106)	− 0.039 (0.104)		0.014 (0.105)	0.058 (0.103)	− 0.019 (0.103)	

The T1b sample size is *n* = 69. FIMLE was used to address missing data; therefore, the sample size for all remaining regressions is *n* = 188 (population size unknown). T1 is prenatal, T2 is when infants were 2–4 months, T3 is when infants were 4–6 months, and T4 is when infants were 10–12 months

*PRE* Prenatal Well-being Program, *SEA* SEACAP Parenting Program, *PPW* Postpartum Well-being Program, *COV* covariates, *1* significant effect of economic strain, *2* significant effect of housing instability, *3* significant effect of number of sessions attended when added to the model in sensitivity analyses

^t^*p* ≤ 0.10; *p < 0.05; **p < 0.01

Regression analyses were used to test whether the prenatal program was related to differences in maternal mental health, mindfulness, parenting, and infant well-being in the early post-partum period, when infants were 2–4 months old (T2) controlling for T1 levels of the corresponding variables and covariates. There were no significant differences on maternal mental health or mindfulness; however, mothers in the prenatal group demonstrated significantly higher baseline RSA. Mothers in the prenatal group demonstrated lower levels of negative control in their interactions with their infants than all other mothers. Infants whose mothers were in the prenatal program demonstrated significantly lower baseline cortisol and a trend toward higher infant HPA-axis reactivity to the still-face condition compared to all other groups.

Regression analyses were used to test whether the three groups differed from each other and from the control group by examining the effects of group status on postpartum post-test assessments (T3) when infants were 4–6 months old, controlling for postpartum pretest (T2) levels of corresponding variables and covariates. With regard to maternal mental health, the postpartum SEACAP parenting group demonstrated significantly lower anxiety compared to all other groups. There were no other significant differences on measures of maternal mental health at post-test. On measures of parenting, the SEACAP group demonstrated higher responsiveness than all other groups. All of the intervention groups demonstrated higher levels of scaffolding behavior at post-test (T3) than the control group. On infant well-being measures, there were no significant differences on observed self-soothing, negative or positive affect, RSA, or cortisol measures at the T3 post-test assessment.

To test for differential effects of the programs at follow-up, the effects of group status on maternal mental health and infant well-being at T4, when infants were 10–12 months old, were examined. There were no significant differences on maternal outcomes. Infants in the SEACAP parenting group demonstrated significantly higher parent-reported effortful control.

We examined whether mindfulness practice was related to participation in the interventions. Participants reported on the extent to which they continued to use specific mindfulness practices at the T4 10–12-month assessment. Participants in the prenatal well-being group reported more frequent use or use of more of the practices (*r* = 0.29, *p* < 0.001), which was not the case for the other groups. Greater use of mindfulness practices was related to higher maternal resilience (*r* = 0.22, *p* = 0.01), but was unrelated to depression or anxiety symptoms.

Sensitivity analyses were conducted to assess the extent to which attendance would impact the results. The pattern of results was very similar when the number of sessions attended was included as a covariate in the regression models. The greater number of sessions attended was related to higher mindfulness and infant baseline RSA at T2, and with higher observed infant self-soothing at T3. The magnitudes of regression coefficients for intervention effects varied, either increasing or decreasing by ≤ 0.13. Most effects that were significant in the main analyses remained significant when the number of sessions attended was included as a covariate. The effect of the postpartum well-being program on increased T3 maternal negative control parenting became significant (*β* = 0.27, *p* < 0.05). The effects of all 3 interventions on T4 maternal resilience became significant (prenatal well-being *β* = 0.21, SEACAP parenting *β* = 0.20, and postpartum well-being *β* = 0.22, all *p* < 0.05). The significant effect of the SEACAP parenting program on T4 infant effortful control became a trend effect (*β* = 0.18, *p* < 0.10).

In some cases, the effects of the covariates were significant. Higher economic strain was related to higher maternal anxiety and lower infant baseline RSA at T2, lower infant self-soothing at T3, and lower effortful control at T4. Housing instability was related to higher maternal depression and lower infant baseline RSA at T2, and higher maternal depression and lower scaffolding at T3.

Finally, we conducted post hoc exploratory analyses to identify potential mechanisms of the intervention effects. When an intervention showed effects on an infant outcome as well as maternal outcome, it was suggestive of specific mechanisms that were explored by examining correlations. First, the prenatal program was associated with lower maternal depression during pregnancy, as well as higher baseline RSA, lower observed negative control parenting, and lower infant baseline cortisol levels at T2, where each of the first three variables could possibly account for the effects of the intervention on infant cortisol levels. The correlations of T2 infant baseline cortisol with those variables were T1 maternal depression (*r* = 0.14, *p* = 0.12), T2 maternal baseline RSA (*r* =  − 0.06, *p* = 0.52), and T2 observed negative control parenting (*r* = 0.21, *p* = 0.02). Similarly, the postpartum parenting intervention was associated with lower maternal anxiety and higher responsive parenting, as well as higher infant effortful control. The correlations of T4 infant effortful control with those variables were T3 maternal anxiety (*r* =  − 0.20, *p* = 0.01) and T3 responsiveness (*r* =  − 0.03, *p* = 0.80). Finally, all three intervention groups showed increases in observed maternal scaffolding behaviors, and mothers’ continued use of mindfulness practices at T4 was correlated with more observed scaffolding behavior at T3 (*r* = 0.26, *p* = 0.003) and with T4 infant effortful control (*r* = 0.23, *p* = 0.004). However, scaffolding was unrelated to infant effortful control (*r* =  − 0.01, *p* = 0.93).

## Discussion

This study evaluated the impact of three perinatal mindfulness-based prevention programs that differed in their timing and target to better understand when and on what targets interventions might impact maternal and infant mental health. The programs were evaluated in a sample of first-time mothers living in low-income contexts to assess whether brief prevention programs can positively impact maternal and infant mental health and potentially be an accessible option for promoting well-being. Program effects were examined on maternal reports of well-being, observed parenting, observed infant emotion regulation, and both maternal and infant physiological indicators of emotion regulation. Overall, there was limited evidence of program effects, likely as a result of the high-risk nature of the sample and the challenges for new mothers to attend in-person groups. Despite these challenges, the prenatal well-being program was related to improved mental health during pregnancy, as well as higher maternal baseline RSA, lower negative control parenting, and lower infant cortisol levels in the early postpartum period, suggesting potential benefits to the infants. In addition, the postpartum parenting program was related to improved maternal sensitivity and lower anxiety, which might be reflected in significant increases in effortful control in infants at 10–12 months.

This study is among the few attempts to assess mindfulness-based programs for a low-income, high-risk group of new mothers. Although the evidence of program effects was somewhat limited given the number of outcomes investigated, the findings were consistent with the hypotheses. Also, it is important to note that this study represents a conservative test of intervention effects, simultaneously testing the effects of the three programs against each other to identify potential specificity of effects. Further, the mothers participating in the study reported experiences of economic strain, housing instability, and high rates of adverse childhood experiences, and the high-risk nature of their contexts might have muted intervention effects. As noted in Table 6, there were significant effects of economic strain and housing insecurity covariates on a number of outcomes. In addition, the brevity of the programs might have provided insufficient intervention doses, and although the programs were brief to increase accessibility, rates of attendance in the postpartum groups were low. In a few cases, the number of sessions attended was related to outcomes. Finally, the study went beyond assessment of maternal reports regarding benefit from the programs, using observational and physiological measures of parenting and emotion regulation, thus providing a more in-depth test of program effects.

Nonetheless, there were several effects of the interventions on maternal mental health, parenting, and infant self-regulation. The mothers who received the prenatal well-being intervention demonstrated lower levels of depression and a trend toward lower anxiety during pregnancy. This finding is consistent with research showing that mindfulness-based interventions can benefit mental health (van Agteren et al., 2021), particularly during pregnancy (Dhillon et al., 2017). It is notable that these benefits did not persist into the early postpartum period when infants were 2–4 months old, suggesting that the transition to parenthood can pose new challenges for parents, and that a more sustained program would be needed through the transition to parenthood. In addition, sustained effects into the postpartum might have been present for those who maintained a mindfulness practice. However, parents who participated in the prenatal program demonstrated higher baseline RSA, indicating better emotion regulation, and lower negative control parenting compared to all other groups when their infants were 2–4 months old. Thus, although there were no sustained benefits to their mental health, the parents who participated in the prenatal intervention might have been better able to manage the challenges of being a new parent.

Notably, there was a significant effect of the prenatal program on lower infant baseline cortisol. This could reflect two possible mechanisms of intervention effects. First, fetal programming of the stress response system has been shown to relate to maternal prenatal stress or mental health (e.g., Harris & Seckl, 2011), and reduced maternal depression in the prenatal intervention group might have resulted in better-regulated HPA-axis activity for infants. Alternatively, infants with parents with lower levels of negative control might also have a better-regulated HPA-axis, consistent with evidence that parental negativity was related to a less regulated HPA-axis (e.g., Zalewski et al., 2012). The latter possible mechanism appears more likely given the significant correlation between maternal negative control and infant baseline cortisol levels in this sample.

Mothers who participated in the postpartum SEACAP parenting intervention demonstrated lower anxiety and higher responsiveness at post-test when infants were 4–6 months old. It is possible that, having received more support for effective parenting that perhaps bolstered their sense of parenting confidence, those mothers were also less anxious as new parents. This is consistent with some evidence that parenting interventions can also reduce parental mental health symptoms in high-risk samples (Booth-LaForce et al., 2023; Rosenblum et al., 2017). Mothers who received the parenting intervention reported greater increases in infant effortful control when their infants were 10–12 months old. This is consistent with evidence that positive parenting behaviors such as warmth and scaffolding promote the development of effortful control in early childhood (e.g., Lengua et al., 2014; Neppl et al., 2020). However, in this study, lower maternal anxiety when infants were 4–6 months, and not responsiveness, was related to later higher infant effortful control, suggesting it is important to examine the potential effects of maternal anxiety on children’s developing self-regulation.

There were some nonspecific intervention effects to note. All parents who received one of the interventions demonstrated more scaffolding behaviors when their infants were 4–6 months compared to mothers in the control group. This effect might reflect benefits of mindfulness programs in general. This is supported by the positive correlation between mothers’ observed scaffolding and their report of continued use of the mindfulness practices. Alternatively, it might reflect mothers’ benefits from having participated in an intervention with other new mothers. Importantly, there were no sustained effects on maternal mental health at 10–12 months postpartum. Given the historical and contextual experiences reported, study participants may have been experiencing fairly entrenched or recurring mood concerns, to which pregnancy and the postpartum made them only more vulnerable (Soares & Zitek, 2008). Nonetheless, in more economically advantaged samples of women at risk for recurrence of depression, similar interventions designed to have protective effects are preferred to pharmacological interventions (Dimidjian & Goodman, 2014), pointing to the need for further efforts to develop and disseminate programs such as those evaluated in this study.

Although one goal of this study was to evaluate relatively brief programs to enhance accessibility of the interventions, poor attendance in the postpartum groups was a significant issue. In some cases, there was a dose effect of the interventions on outcomes, which might imply that better rates of attendance would potentially lead to more robust intervention effects. Post-intervention feedback from participants indicated that parents who were able to attend the sessions appreciated the social support provided by the group of parents, whereas those who attended few, if any, sessions indicated that attending in person with their infants was challenging for numerous reasons including infant and mother illness, changing work schedules, and concerns about transporting their infants (Calhoun et al., under review). Even prior to the COVID-19 pandemic, new mothers reported the preference for the ability to access a group intervention program online to benefit from both the social support of other new mothers and easier access to the program. This might be particularly true for this sample of mothers who were all living in a low-income context, whereas when such programs are offered in low-risk samples, poor attendance has not been an issue (e.g., Sbrilli et al., 2020; Vieten et al., 2018). A critical direction is to identify a structure and mode of delivery that increases accessibility of such programs.

### Limitations and Future Directions

It is important to note several limitations of this study. Participants were not randomly assigned to groups making it possible that pre-existing differences account for observed intervention effects. In fact, the control group reported higher levels of economic strain and housing insecurity. However, given that group assignment was based on estimated due dates, little systematic bias was expected to be introduced by nonrandom group assignment. Also, attendance at the postpartum groups was low, highlighting the challenges of accessibility of such programs for new parents in a low-income context. Parents indicated numerous challenges with attending, as mentioned above. In addition, the sample sizes within each group were somewhat small, resulting in insufficient power to detect small effects sizes. Further, the groups were disproportionate in size which potentially violates the assumption of equal variances and increases the likelihood of type-I errors (Rusticus & Lovato, 2014). Despite the study’s limitations, it had several strengths that speak to promising future directions. The inclusion of low-income mothers who were racially and ethnically diverse suggests that the interventions can be acceptable in diverse communities. Also, the use of observational and physiological measures ensured that the findings were not based solely on self-report, and the longitudinal design that included baseline and follow-up assessments allowed for tests of the timing and maintenance of intervention effects.

Significant effects suggest there could be value in mindfulness-based interventions offered both prenatally and postpartum, particularly if they are offered in a way that facilitates attendance for parents living in high-risk contexts. Such programs should be offered and evaluated in the context of other critical support to new parents, including stable housing, food and income security, paid parental leave, and high-quality childcare, all factors known to support maternal and infant mental health. In a context of such support, mindfulness-based prenatal well-being and postpartum parenting preventive interventions may promote maternal mental health and effective parenting that, in turn, will support children’s development of self-regulation and social, emotional, and behavioral well-being in early childhood.

## Data Availability

Data are available upon reasonable request from the first author.
